# Lignan Content in Cereals, Buckwheat and Derived Foods

**DOI:** 10.3390/foods2010053

**Published:** 2013-02-07

**Authors:** Alessandra Durazzo, Maria Zaccaria, Angela Polito, Giuseppe Maiani, Marina Carcea

**Affiliations:** National Research Institute on Food and Nutrition (INRAN), Via Ardeatina 546, 00178, Rome, Italy; E-Mails: zaccaria@inran.it (M.Z.); polito@inran.it (A.P.); maiani@inran.it (G.M.); carcea@inran.it (M.C.)

**Keywords:** lignans, cereal grains, cereal based foods, buckwheat, database

## Abstract

Cereal foods are a fundamental part of a balanced diet and several studies have assigned to wholemeal cereal products a protective role in human health, due to their content of bioactive compounds. Within the phytochemicals, lignans are of increasing interest for their potential anticarcinogenic, antioxidant, estrogenic and antiestrogenic activities. The aim of this work is to contribute to the updating of food lignan databases by providing the profile and the amount of lignans in cereals, buckwheat and several cereal based foods commonly consumed in human diets. Values were taken from published papers. Items were divided in different groups, namely *grains*, *brans and flours*, *bread*, *cereal staple foods*, *breakfast cereals* and *other cereal products*, and values for secoisolariciresinol, matairesinol, pinoresinol, lariciresinol are given. For example, the total average values for the mentioned lignans in grains ranged between 23 μg/100 g and 401 μg/100 g dry weight. The contribution of each single lignan molecule to the total value of lignans appears to be different for every cereal species. Lignan content and typology in processed foods depends on the raw materials used, their degree of refinement and on processing conditions.

## 1. Introduction

Cereal food products are a fundamental part of a balanced diet and recently several studies have assigned to cereal grains and to wholemeal cereal products in particular a protective role in human health due to their content of bioactive compounds. Bertram *et al.* [1] conclude that the main protective foods are represented by fiber- and lignan-rich whole-grain cereals, beans, berries, nuts and various seeds.

Within the group of the so called phytochemicals, the phenolic compounds named lignans are attracting the interest of food chemists and nutrition researchers alike. Lignans are vascular plant secondary metabolites, which are attributed a wide range of physiological functions and beneficial properties [2,3,4].

Lignans belong to the group of diphenolic compounds derived from the combination of two phenylpropanoid C6–C3 units at the β and β′ carbon atoms. They have a chemical structure like the 1,4-diarylbutan. They are derived from the shikimic acid biosynthetic pathway [5,6,7]. They are optically active compounds and may exist as two enantiomers, *i.e.*, the right- and left-handed forms [8,9]. Numerous structurally different forms of lignans exist, even if their molecular backbone consists only of two phenylpropane units.

Lignans are contained in edible plants where they occur free or bound to sugars [10]. Several hundred lignans have been discovered in different parts of various plants, including wooden parts, roots, leaves, flowers, fruits and seeds. The plant lignans most commonly detected in foods are lariciresinol, matairesinol, pinoresinol and secoisolariciresinol [11,12]. Other lignans such as medioresinol, syringaresinol, sesamin were reported in various kinds of foods [13,14,15,16]. However, it is important to point out that all lignans present in all foods have never been analysed in any study so far. The main sources of dietary lignans are oilseeds (e.g., flax, soy, rapeseed and sesame), whole-grain cereals (e.g., wheat, oats, rye and barley), legumes and various vegetables and fruits (particularly berries) [11,12,13,14,15,16]. Amongst edible products flaxseed and sesame seeds are rich sources of lignans [11,15,17,18,19], but wood knots in coniferous trees, especially Norway spruce, are the most concentrated lignan sources known so far [20].

Some of the ingested plant lignans are deglycosylated and partly converted to the mammalian lignans enterodiol and enterolactone by colonic bacteria: enterodiol is readily oxidized to enterolactone [21,22,23,24]. These metabolites are then absorbed in the colon and conjugated with glucuronic acid or sulfate in the liver. Some of the metabolites may also undergo enterohepatic circulation. Lignans are excreted in bile and urine as conjugated glucuronides and in feces in the unconjugated form [25,26].

Enterodiol and enterolactone, which are generally called enterolignans due to their colonic origin, have a similar structure to the human hormone estrogen and so may have estrogenic/anti-estrogenic effects. Several epidemiological studies have shown a potential protective effect of the enterolignans or of a lignan-rich diet against the development of various diseases, particularly hormone dependent cancer and cardiovascular diseases [27,28,29,30,31,32,33,34,35,36,37]. The health effect of lignans varied depending on the particular lignan type. It is well known that the physiological activities of compounds are affected by their molecular structures.

The knowledge of the dietary intake of lignans is most notably needed to understand the relationship between a lignan-rich diet and probable prevention of various diseases such as hormone-related cancers, heart diseases, menopausal symptoms and osteoporosis. For this reason, a complete and comprehensive database on the content of lignans in foods is needed [38]. However, the available information has some limitations: many studies focused only on a few compounds, the structural diversity of the compounds, the large number of dietary sources, the large variability in content for a given source, the diversity of extraction techniques and analytical methods used, and, in some cases, the lack of suitable analytical methods [39].

Accurate information on dietary lignan in foods is crucial to determine exposure and to investigate health effects *in vivo*. Recently, more analytical data on lignans have become available and they could be used to expand food composition databases [11,12,13,14,15,16,40,41,42,43].

The aim of this work is to contribute to the updating of food lignan databases in general, focusing in particular on cereal grains and cereal based foods by gathering and systematizing available data coming from scientific publications. Our list can be a valuable tool for nutritionists, dietitians, medical doctors and scientists in general to estimate the human dietary exposure to lignans coming from the consumption of cereal based foods and in evaluating the effects of cereals consumption in epidemiological studies. Moreover, it is a document stating the state-of-the-art on this topic.

## 2. Experimental Section

### 2.1. Evaluation and Selection of Available Lignan Data

An extensive bibliographical search was conducted using the following keywords: lignans, secoisolariciresinol, lariciresinol, pinoresinol, matairesinol, medioresinol, syringaresinol, cereals and names of individual cereals, cereals based foods and names of individual foods. Having laboratory experience in the determination of lignans we were also aware that the extraction method from the food matrix is an important issue: acid hydrolysis effectively breaks the ester linkages and the glycosidic bonds, but may also affect the molecular structure, causing interconversions between lignans. On the other hand, alkaline hydrolysis is not effective enough in some instances, particularly in strong matrices such as fiber-rich foods, and can lead to underestimation of matairesinol [4,13]. 

Therefore, an extraction strategy used to determine the amount of dietary lignan in foods was chosen as preferable and publications using that method were selected in order to assure a fair data comparison. The method by Penãlvo *et al.* [15] that adopts alkaline hydrolysis as the step prior to enzymatic hydrolysis, was chosen. Under alkaline conditions, ester-linked oligomers of lignan are hydrolysed to give the lignan monomer. This method also incorporates isotope dilution to ensure correct accuracy and precision, introducing the utilization of individual stable ^13^C_3_-labeled lignans. 

Analytical values using isotope dilution as internal standards and either gas or liquid chromatography-mass spectrometry [11,13,14,15,44,45] were considered to be preferable because this was the most sensitive analysis available, having the lowest level of detection. However, values obtained by HPLC were also considered, even if HPLC is less selective and sensitive for the detection of low concentrations of the compounds under study.

### 2.2. Database Construction

For easiness of comparison foods were grouped in seven different categories, namely *grains*, *brans and flours*, *bread*, *cereal staple foods*, *breakfast cereals* and *other cereal products*. Grains included barley (*Hordeum vulgare* L.), buckwheat (*Fagopyrum esculentum* Moench.), durum wheat (*Triticum durum* Desf.), emmer (*Triticum dicoccon* Shrank.), maize (*Zea mais* L.), oat (*Avena sativa* L.), rice (*Oryza sativa* L.), rye (*Secale cereale* L.), soft wheat (*Triticum aestivum* L.), spelt (*Triticum spelta* L.) and triticale (*× Triticosecale* Wittm).

The average and range values were reported for secoisolariciresinol, matairesinol, lariciresinol, pinoresinol, whether the reported values were on a wet or dry basis, together with other useful information such as origin of samples, genetic background, origin, composition and treatment. Despite syringaresinol and medioresinol are present in cereals and cereal based foods, few data on these lignans are available in the literature [13,14,15,16], as a consequence, sometimes, of restricted standards availability.

## 3. Results and Discussion

Cereals, whole grain cereals in particular are an important source of lignans in the human diet [11].

The data presented here (Table 1) are a comprehensive and systematized assessment of lignans in cereals and cereal based products for a more accurate determination of exposure to dietary lignans from foods which are commonly consumed by the population in different countries. For ease of consultation, items are grouped in different categories and are listed in each group in alphabetical order.

**Table 1:** Database of lignans (secoisolariciresinol, matairesinol, lariciresinol, pinoresinol) composition in cereals, buckwheat and derived foods *.

Foods	Seco	Mat	Lari	Pino	Unit	Origin, composition and/or treatment	Reference
*Grains*							
Barley	30	3	85	72	μg/100 g wet basis	Local markets; dehulled	Penãlvo *et al.* [15]
Barley	28	n.d.	132	45	μg/100 g dry weight	Italian farms; 2 cultivars; dehulled	Durazzo *et al.* [40]
Buckwheat	131	1	362	92	μg/100 g wet basis	Local markets	Penãlvo *et al.* [15]
Durum wheat	n.d.	n.d.	76	n.d.	μg/100 g dry weight	Italian farms; 2 cultivars	Durazzo *et al.* [40]
Emmer	29	n.d.	104	n.d.	μg/100 g dry weight	Italian farms; 2 cultivars	Durazzo *et al.* [40]
Maize	12	n.d.	11	0	μg/100 g dry weight	Italian farms; 3 cultivars	Durazzo *et al.* [40]
Millet	67	3	20	85	μg/100 g wet basis	Local markets	Penãlvo *et al.* [15]
Oat	19	71	183	194	μg/100 g wet basis	Local markets	Penãlvo *et al.* [15]
Oat	n.d.	n.d.	97	304	μg/100 g dry weight	Italian farms; 2 cultivars; dehulled	Durazzo *et al.* [40]
Oat	6–13	0–104	340–599	214–683	μg/100 g wet basis	Fifty-five spring oat samples of 5 different cultivars	Smeds *et al.* [14]
Rice	15	n.d.	128	29	μg/100 g dry weight	Italian farms; 3 cultivars	Durazzo *et al.* [40]
Rye	38	27	324	381	μg/100 g wet basis	Local markets	Penãlvo *et al.* [15]
Rye	25	n.d.	100	n.d.	μg/100 g dry weight	Italian farms; 2 cultivars	Durazzo *et al.* [40]
Rye	10–29	18–45	76–177	176–313	μg/100 g wet basis	Twenty-eight winter rye samples of 6 different cultivars	Smeds *et al.* [14]
Soft wheat	n.d.	n.d.	58	n.d.	μg/100 g dry weight	Italian farms; 2 cultivars	Durazzo *et al.* [40]
Soft Wheat	35	3	62	37	μg/100 g wet basis	Local markets	Penãlvo *et al.* [15]
Soft Wheat	20–43	n.d.	45–95	53–83	μg/100 g wet basis	Seventy-three spring wheat samples of 9 different cultivars	Smeds *et al.* [14]
Spelt	26	n.d.	83	n.d.	μg/100 g dry weight	Italian farms; 2 cultivars	Durazzo *et al.* [40]
Triticale	n.d.	n.d.	58	n.d.	μg/100 g dry weight	Italian farms; 2 cultivars	Durazzo *et al.* [40]
*Brans and flours*							
Durum wheat bran	n.d.	n.d.	220	181	μg/100 g dry weight	Two cultivars	Durazzo *et al.* [41]
Soft wheat bran	370	n.d.	459	386	μg/100 g dry weight	One cultivar	Durazzo *et al.* [41]
Soft wheat	31	0	140	38	μg/100 g wet basis	Supermarkets; wholemeal	Milder *et al.* [11]
Soft wheat	0	0	18	9	μg/100 g wet basis	Supermarkets; whiteflour	Milder *et al.* [11]
Soft wheat	16	n.d.	34	n.d.	μg/100 g dry weight	Supermarkets; whiteflour	Durazzo *et al.* [43]
*Bread*							
Currant/raisin	9	7	79	9	μg/100 g wet basis	Two supermarkets and a local bakery	Milder *et al.* [11]
Flaxseed (whole)	11,845	26	220	383	μg/100 g wet basis	Two supermarkets and a local bakery	Milder *et al.* [11]
Flaxseed	7208	0	29	2	μg/100 g wet basis	Dempsters; pre-sliced	Thompson *et al.* [12]
Multi-grains	6163	19	185	377	μg/100 g wet basis	Two supermarkets and a local bakery	Milder *et al.* [11]
Multi-grains	4770	1	10	4	μg/100 g wet basis	Dempsters; pre-sliced	Thompson *et al.* [12]
Oat	7	0	4	11	μg/100 g wet basis	Wheat and oats with honey; pre-sliced	Thompson *et al.* [12]
Rye (dark type)	13	14	122	172	μg/100 g wet basis	Two supermarkets and a local bakery	Milder *et al.* [11]
Rye (light type)	16	12	111	163	μg/100 g wet basis	Two supermarkets and a local bakery	Milder *et al.* [11]
Rye	122	0	11	9	μg/100 g wet basis	Jagdschnitten hunter style, Dimpflmeier; pre-sliced	Thomson *et al.* [12]
Rye	33	4	47	44	μg/100 g wet basis	Local markets in Tokio; (50% rye)	Penãlvo *et al.* [16]
Rye	7	1	18	16	μg/100 g wet basis	Local markets in Tokio; (30% rye)	Penãlvo *et al.* [16]
Sesame	3	0	8	42	μg/100 g wet basis	Dempsters; pre-sliced	Thompson *et al.* [12]
Wheat (whole type)	3	0	5	1	μg/100 g wet basis	Original 100%, Dempsters; pre-sliced	Thompson *et al.* [12]
Wheat (whole type)	15	0	73	33	μg/100 g wet basis	Two supermarkets and a local bakery	Milder *et al.* [11]
Wheat (refined type)	17	0	38	28	μg/100 g wet basis	Two supermarkets and a local bakery	Milder *et al.* [11]
Wheat (white type)	0	0	11	7	μg/100 g wet basis	Two supermarkets and a local bakery	Milder *et al.* [11]
Wheat (white type)	1	0	2	1	μg/100 g wet basis	Enriched, Wonder; pre-sliced	Thompson *et al.* [12]
*Cereals staple foods*							
Couscous (cooked)	2	n.d.	0	n.d.	μg/100 g wet basis	President’s choice; cooked in water	Thompson *et al.* [12]
Macaroni (cooked)	4	0	7	5	μg/100 g wet basis	White, boiled	Milder *et al.* [11]
Rice (cooked)	3	2	28	7	μg/100 g wet basis	Supermarket; whole grain, boiled	Milder *et al.* [11]
Rice (cooked)	0	0	7	0	μg/100 g wet basis	Supermarket; white, boiled	Milder *et al.* [11]
Rice (cooked)	0	0	3	1	μg/100 g wet basis	White, converted long grain, Uncle Ben’s; cooked in water	Thompson *et al.* [12]
Semolina pasta (raw)	22	n.d.	26	27	μg/100 g dry weight	Market; three different brands	Durazzo *et al.* [41]
*Breakfast cereals*							
*Brand 1*	7	n.d.	79	69	μg/100 g dry weight	Market; cereals 48.4% (whole oat flour 35.8%; maize flour), wheat germ	Durazzo *et al.* [41]
*Brand 2*	20	n.d.	97	131	μg/100 g dry weight	Market; whole cereals (54%) (flour of whole oat, whole rice, whole wheat), cereal agglomerate (19%), oat bran, barley malt	Durazzo *et al.* [41]
*Brand 3*	n.d.	n.d.	99	187	μg/100 g dry weight	Market; cornflakes and bran (31.5%), toasted oatmeal (30%), rice aggregate and bran (25%), sugar-coated barley flakes (9%), almonds (4.5%)	Durazzo *et al.* [41]
Cheerios	9	1	3	0	μg/100 g wet basis	General Mills	Thompson *et al.* [12]
*Muesli*	17	0	250	497	μg/100 g wet basis	Jordans, crunchy	Milder *et al.* [11]
*Muesli*	13	0	120	210	μg/100 g wet basis	Albert Heijn, basic	Milder *et al.* [11]
*Muesli*	17	0	63	129	μg/100 g wet basis	Edah, crunchy	Milder *et al.* [11]
*Oatmeal*	1	0	4	2	μg/100 g wet basis	Dempsters; quick cooking, boiled in water	Thompson *et al.* [12]
*Raisin Bran*	15	0	17	1	μg/100 g wet basis	Kellogg’s	Thompson *et al.* [12]
*Other cereal products*							
*Compressed puffed rice*	23	n.d.	82	22	μg/100 g dry weight	Market; white rice and dehulled rice	Durazzo *et al.* [41]
*Puffed barley*	26	n.d.	143	48	μg/100 g dry weight	Market; whole barley	Durazzo *et al.* [41]
*Wholegrain biscuits*	28	n.d.	25	23	μg/100 g dry weight	Market; wheat flour (51%), barley flakes (3%), rye flakes (1.8%), rice flour (1.7%), oatmeal (1.3%), maize flour (1.2%), wheat malt	Durazzo *et al.* [41]
*Granola bar*	3	2	8	14	μg/100 g wet basis	Nature Valley, with almond	Thompson *et al.* [12]

* Seco = Secoisolariciresinol; Mat = Matairesinol; Lari = Lariciresinol; Pino = Pinoresinol; n.d. = not detectable.

Among grains, Durazzo *et al.* [40] showed that dehulled oat and barley reached the highest values (by summing up the analysed lignans) amongst the 10 reported cereal species, 401 and 206 μg/100 g dry weight, respectively. A great variability was observed with regard to lignan typology and content also within the same species.

In fact, the content of some lignans and the degree of esterification of the same lignan glycosides may vary within the same cereal species as a consequence of different growing conditions, geographic location, climate, and genetic characteristics [11,13,14]. In fact, agronomic, environmental and post-harvest factors influence, in different ways, not only different classes of chemical compounds but also structurally different compounds belonging to the same chemical subgroup [46]. However, in most of the studied grains the predominant compound amongst the reported lignans was lariciresinol [14,15,40].

Many grains are consumed daily in a number of products from around the world. The health benefits of cereal products are an emergent part of the health related food market. Grains undergo different kinds of processing to obtain desirable products with optimized flavor, color, texture and appearance as well as shelf life. Few results are reported in the literature regarding the effects of cereal processing technologies such as milling, baking, extrusion, etc. on lignans content in most foods. 

Regarding flours, considering that lignans are mainly located in the grain outer layers [13,47,48], it is understandable that wholemeal flour is richer in lignans than refined flours. The same difference was observed in whole wheat bread with respect to refined wheat bread in both the investigations of Milder *et al.* [11] and Thompson *et al.* [12]. 

Miur and Westcott [49] reported that secoisolariciresinol diglucoside was stable in the bread making process. In particular, it could withstand the higher temperatures in the core during baking. Simbalista *et al.* [50], by investigating the effect of bread making on a product containing flaxseed, have shown that, after baking, 89% of the original lignan content was kept in bread.

Among bakery products, besides those containing flaxseed and multi-grains, rye bread represents a good source of all different lignans [11,12,16]. 

Regarding breakfast cereals, Milder *et al.* [11] found a range between 209 and 764 μg/100 g wet basis considering the sum of lariciresinol, secoisolariciresinol and pinoresinol. Durazzo *et al.* [41] found a range of total lignan content (same as above) from 154 to 286 μg/100 g dry weight. Lariciresinol and pinoresinol were found at higher concentrations with respect to secoisolariciresinol in most of the items. Obviously, the different raw materials used and their degree of refinement are mainly responsible for the different lignan concentrations in breakfast cereals but, in general, they are a good source of lignans.

As regards the cereals staple foods group, pasta is recognized all over the world as an ingredient of traditional meals, especially in the Mediterranean regions [51]. Recent statistics indicate that 26 kg/person of pasta are yearly consumed in Italy [52]. In raw Italian semolina pasta, total lignan content (*i.e.*, the sum of secoisolariciresinol, lariciresinol and pinoresinol) was reported to be 76 μg/100 g dry weight [41]. The concentration of lignans in pasta is relevant considering that pasta is a staple food in the diet of some populations and it is consumed daily.

Pasta undergoes cooking before consumption so it is also important to consider the influence of domestic cooking on the lignans present in the raw pasta. Milder *et al.* [11] reported for cooked macaroni, cooked white rice and cooked whole rice values of total lignan content of 16, 7 and 40 μg/100 g wet basis, respectively. 

As regards *other cereal products*, puffed barley represents a good source of lignans and the most representative component amongst the identified lignans is lariciresinol [41]. The level of total lignans (*i.e.*, the sum of secoisolariciresinol, lariciresinol and matairesinol) in puffed barley matched that observed in dehulled barley grains. So, again, the raw material and its degree of refining determines mainly the amount of bioactive substances, lignans in particular, which are found in raw processed foods.

This database, which is limited and can be updated as new data become available, can nevertheless for the time being be useful for several purposes such as understanding the potential effects of specific lignans in epidemiological studies or estimating the human dietary exposure to lignans coming from cereal based foods.
